# Implementation and sustainability of mentorship for public health student success

**DOI:** 10.3389/fpubh.2025.1657044

**Published:** 2025-10-23

**Authors:** Stephanie A. Grilo, Michael A. Joseph, Goleen Samari

**Affiliations:** ^1^Heilbrunn Department of Population and Family Health, Mailman School of Public Health, Columbia University, New York, NY, United States; ^2^Mailman School of Public Health, Columbia University, New York, NY, United States; ^3^Department of Population and Public Health Sciences, Keck School of Medicine, University of Southern California, Los Angeles, CA, United States

**Keywords:** mentorship, indigenous and people of color, first generation, public health education, advising

## Abstract

Historically underrepresented and marginalized (URM) graduate students in public health, including students of color, first-generation, and international students, often face structural inequities such as limited representation among faculty, insufficient professional networks, and lack of structured mentorship. In response, the Mentoring of Students and Igniting Community (MOSAIC) program was established in 2019 at Columbia University’s Mailman School of Public Health. MOSAIC aims to provide structured, equity-centered mentorship designed to enhance academic success, professional development, and community-building for URM students. This manuscript outlines the implementation and evolution of MOSAIC over 5 years, highlighting key processes in its design, preparation, implementation, and sustainability. The program uniquely integrates anti-racist and anti-oppressive pedagogical frameworks, prioritizes student-driven programming, and involves committed, compensated faculty mentors from diverse backgrounds. Initial evaluations demonstrate positive outcomes, including improved student satisfaction, sense of belonging, and overall quality of life. The sustainability of MOSAIC was achieved through strategic actions such as securing institutional funding, fostering administrative buy-in, recruiting faculty champions, and collaborating with existing campus resources. MOSAIC’s substantial growth from 26 participants in its inaugural year to over 450 by 2024 underscores its effectiveness and the broader institutional commitment to inclusive excellence. This paper uses the implementation and sustainability of MOSAIC at Columbia Mailman to show other public health institutions aiming to replicate and sustain mentorship programs how they can support URM students but also foster institutional transformation toward equity and inclusion.

## Introduction

Historically underrepresented and marginalized students (URM), including students of color, first-generation, and international students, face systemic barriers, including a lack of representation among faculty, limited professional networks, reduced access to the hidden curriculum, and an absence of structured support mechanisms, particularly in graduate school (1–4). These students may benefit the most from a mentoring relationship (3, 5, 6). However, mentorship is a critical yet often overlooked component of student success in public health education (7–10). Addressing these gaps requires intentional, equity-centered mentorship models that not only provide academic guidance but also affirm students’ identities and lived experiences within historically exclusionary institutional spaces.

Even as the need for robust mentorship becomes increasingly clear, institutional efforts to support URM students often stop at access, without addressing the structural inequities that continue to shape their academic experiences. Over the past decade, predominantly White institutions (PWIs) have emphasized increasing URM student enrollment (11), but have not placed comparable emphasis on supporting these students once they arrive, especially as they navigate majority-dominated academic fields (12, 13). As a result, a large body of evidence indicates that URM graduate students, particularly those in STEM fields report feeling invisible, isolated, and undervalued, not only as students, but throughout their careers (14, 15). Lack of access to relatable faculty role models can diminish students’ satisfaction, self-efficacy, engagement, and academic achievement (16, 17). For URM students, faculty mentoring can be transformative, enhancing both graduate education and career development (7).

Public health institutions have long emphasized commitments to social justice and health equity; however, these values are not always reflected in institutional practices, particularly when it comes to supporting URM graduate students. Despite the field’s stated ideals, many schools of public health lack structured, equity-centered mentorship programs and have been slow to adopt anti-oppressive pedagogical approaches that explicitly address power, privilege, and structural inequities within academia (9, 18–20). Mentoring has been widely recognized as a high-impact practice that improves student persistence, retention, and graduation rates, and also offering professional development benefits for faculty and staff (8, 21, 22). Yet, recent research highlights persistent gaps in student mentorship structures in programs and schools of public health (9). While previous studies have focused on how mentors are trained and supported (23–25), there is little guidance on implementing structured, faculty-to-student group mentorship programs specifically tailored to the needs of URM and first-generation students in public health.

In response to this gap, the Mentoring of Students and Igniting Community (MOSAIC) program was founded in 2019 at Columbia University’s Mailman School of Public Health to provide intentional, faculty-to-student mentorship rooted in equity, community-building, and professional development (9, 26). Designed to meet the unique needs of URM and first-generation students, MOSAIC has expanded rapidly, from serving 26 students in 2019 to over 450 participants by 2024. Evaluation data suggest the program is associated with increased graduate school satisfaction, a stronger sense of belonging, and improved quality of life (26). These promising MOSAIC outcomes reflect the growing call within public health education to move beyond symbolic commitments and embed anti-oppressive and anti-racist strategies into the core functions of teaching, mentorship, and institutional culture (10, 18, 27–29).

To contribute to this growing body of work and address existing implementation gaps, this study focuses on the development and sustainability of the MOSAIC program at Columbia Mailman School of Public Health. This paper examines the process of implementing MOSAIC at Columbia Mailman in 2019 and traces the program’s evolution and sustainability over a five-year period. The primary goal of the paper is to give other schools and programs of public health insights into what it takes to implement and sustain MOSAIC in ways that are durable, equity-driven, and institutionally transformative.

## MOSAIC at Columbia Mailman: program design and implementation

**Figure 1:**
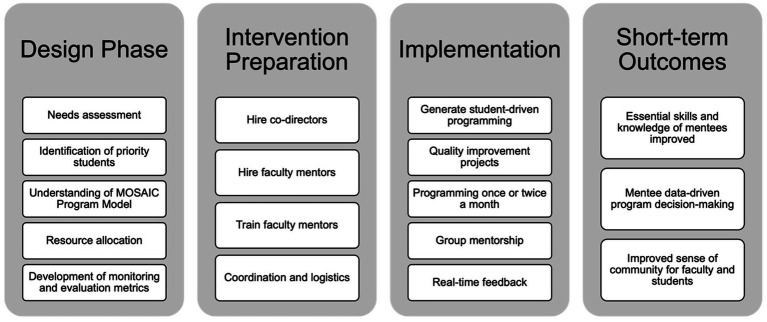
MOSAIC design and implementation process.

MOSAIC was developed through a collaborative, student-centered process that included faculty and student input to ensure the program addressed real and perceived barriers in public health mentorship. The implementation of the MOSAIC program at Columbia University in 2019 involved a structured, multi-phase approach, encompassing four main stages: the design phase, intervention preparation, implementation, and short-term outcomes (Figure 1).

In the design phase, we began by conducting a thorough needs assessment at the Columbia University Mailman school of public health to identify gaps in support and resources for BIPOC and first-generation graduate public health students. Academic advising existed (and continues to exist) for students in their academic departments. Advising includes guidance on course registration, certificate selection, academic concerns and more and is critical to graduate student success. However, BIPOC and first-generation students expressed a lack of community as well as limited connections to BIPOC and first-generation faculty. These students also indicated a need for help navigating the institution and access to available resources and a setting and group where they could raise concerns and questions while practicing professional development skills. Mentors can best help students of color and first-generation students at the beginning of their graduate careers by encouraging a realistic understanding of their work in the context of a larger, sustaining set of goals (20). Thus, we aimed to focus on master’s students from orientation throughout their graduate journey. This initial phase also included establishing a comprehensive understanding of the MOSAIC program model, which draws upon five primary pillars (9), and deciding on the approach we wanted to use to design MOSAIC. MOSAIC’s approach was rooted in anti-racism and anti-oppression pedagogy (AOP). AOP has been defined in multiple ways and generally refers to teaching about oppression, the systems that contribute to oppression, and the disruption of oppression (18, 30). AOP requires one to unlearn or relearn what was previously known about oppression and space is required for learners to work through this process (31). AOP is not commonly used in the health profession and when it is used, the operationalization and application varies widely (19). MOSAIC integrates AOP by providing space for faculty and students to come together about topics related to oppression, student experiences, and public health. Research shows that students appreciate meaningful experiences with faculty that normalize struggle and failure by promoting a growth mind-set, validating student competence and potential, and opening discussion about racialized and gendered dynamics in academia (32).

Faculty mentorship is a foundational element, with faculty committing to ongoing mentorship relationships with students, offering academic guidance, career development support, and professional networking opportunities. The faculty mentors are expected to participate in monthly MOSAIC faculty meetings to help to design the program of events, attend as many events as they can, and hold monthly office hours for MOSAIC students (Table 1). MOSAIC recognizes that in addition to guiding public health careers, providing advice and access to resources, and advocating for their mentees, inclusive mentors readily acknowledge their mentees’ identity, validate their backgrounds and accomplishments, and provide supportive environments to prevent isolation by promoting cultural awareness and sensitivity. This is accomplished through intentional community building that emphasizes trust and rapport, creates space for listening sessions during challenging times or in response to serious events, acknowledges and validates experiences such as imposter syndrome, and connects mentees with supportive resources. For MOSAIC mentors, transparency and validation are key tenants of the approach. Specifically, mentors are encouraged to speak openly about their own experiences and backgrounds, connect challenges to their identities, and share key institutional knowledge with MOSAIC students. Both workshops and informal interactions are used to encourage open dialogue, normalize challenges, and strengthen mentees sense of inclusive and supportive spaces.

**Table 1:** MOSAIC mentor responsibilities and mentorship values and principles.

MOSAIC mentor responsibilities	Mentorship values and principles
Participate in MOSAIC faculty meetings and collaborative program design.	Commitment to justice: sustain institutional transformation toward equity and inclusive excellence.
Attend program events to ensure access and availability and hold regular office hours.	Anti-oppression and inclusivity: validate students’ identities and lived experiences, foster culturally responsive spaces.
Create safe spaces for open dialogue, listening sessions, and processing challenging events.	Trust and reciprocity: prioritize relational engagement, mutual respect, and shared growth.
Share institutional knowledge, including the “hidden curriculum” and navigation strategies.	Transparency and validation: openly share personal experiences, normalize struggle, and affirm mentees’ potential.
Provide academic guidance and career development support (coursework, research, professional pathways).	Equity and anti-racism: intentionally confront structural racism, bias, and inequities within academia.
Facilitate professional networking and connect students to faculty, alumni, and practitioners.	Community-building: cultivate belonging, reduce isolation, and promote peer support.

Although the case has been made that mentors can be and have been effective even with mentees whose racial backgrounds differ from their own, the amount of time, effort, dedication, and knowledge, and skill it takes to be effective mentors with mentees who are already isolated in PWIs is quite high. Thus, mentors, often, do not have the requisite training to provide URM mentees with the kind of mentorship needed to navigate racial barriers and thrive in an isolating academic environment (33). Studies show that mentoring program practitioners should elevate their communication with mentees, either directly or through mentors, to learn more about the mentee experience and their dispositions toward mentoring (34). Thus, MOSAIC intentionally includes faculty of color or first-generation faculty and compensates faculty for their involvement as faculty mentors.

Community building is another critical aspect, as MOSAIC fosters a sense of belonging through cohort-based mentorship, social events, and peer support networks. To help students successfully navigate institutions, the program provides structured guidance on coursework, research, administrative processes, and funding opportunities. Professional development is prioritized through tailored workshops, guest speaker panels, and skill-building sessions to prepare students for careers in public health. A sample semester schedule of events can be found in Table 2 and demonstrates the mix of formal and informal events that we put together each semester.

**Table 2:** Example types of MOSAIC programming in an academic year.

Event	Description	Semester	Type
Orientation	MOSAIC introduction for first year master’s students	Summer	Formal
Welcome event	First event of the year: welcoming back second year students and new first year students and planning programming for the year with student input	Fall	Informal
Faculty meet and greet	MOSAIC students meet and interact with faculty and administrators across the school	Fall	Formal
Resume and workshop with career services	A co-sponsored event with Career Services for students to work on their resume and pitch before the school wide career fair	Fall	Formal
Writing specialists support	A co-sponsored event with the writing specialists through academic support for students to get help with major writing assignments	Fall	Informal
Potluck/multicultural day	An informal get together for students and faculty to form community	Fall	Informal
Day of service	A school wide event where MOSAIC partners with community organizations around NYC and provides volunteer experiences for staff, faculty and students	Fall	Formal
Wellness kits	A study break to do some crafting and form community	Fall	Informal

The intervention preparation phase involved critical operational tasks, beginning with the hiring of program co-directors, who were specifically faculty of color or first-generation faculty to foster identity alignment and relatability for students. In 2019, the program co-directors founded and launched the program with no financial compensation and only themselves serving as faculty mentors. Both co-founders identify as faculty of color and had been approached by countless students with questions about navigating graduate school and with comments about feeling a lack of community. Through these individual student meetings, they recognized that the same questions and experiences were being brought to them and how it would be much more effective to develop a group mentorship model for Mailman students. MOSAIC was prepared to serve one department at the Mailman School of Public Health, but it remained open to students from any department within the school. Significant attention was given to coordination and logistics, ensuring smooth and effective program delivery. In the first year, this fell to the program co-directors, but in subsequent years, MOSAIC had administrative support (such as help booking rooms and ordering food and supplies for meetings) as well as support from a MOSAIC student research assistant.

The third phase, implementation, marked the practical rollout of the program. Central to implementation was student-driven programming, which placed mentees’ voices and needs at the center of program activities. In its inaugural year (2019), MOSAIC successfully engaged 26 students, laying the groundwork for its subsequent expansion and broader institutional impact. During the first MOSAIC meeting, students are asked to brainstorm ideas for types of events and programming they would benefit from. These student generated ideas are then used to create programming for the year. MOSAIC meets once or twice a month and in the first year, 2019, MOSAIC consistently met twice a month. Perhaps, most surprising was the student requests for more informal mentorship opportunities. The model then included one formal event per month and one informal event where students could interact with faculty in a casual manner. Regular quality improvement projects were integrated to continually enhance program responsiveness and effectiveness. MOSAIC programming occurred approximately once or twice monthly, providing consistent opportunities for engagement. Additionally, the program featured structured group mentorship sessions, creating spaces for collective support and peer learning. MOSAIC is not an individual mentorship program so students are not matched with one mentor. Instead, all faculty affiliates attend the events and hold monthly office hours. Students are free to attend all office hours for the faculty involved and interact with all faculty at MOSAIC events. Real-time feedback from students was actively collected to allow continuous adaptation and responsiveness to participant needs.

Finally, the short-term outcomes phase identified initial impacts of MOSAIC during its first year. These outcomes included measurable improvements in mentees’ essential academic and professional skills. Data collected from mentees directly informed ongoing programmatic decision-making, ensuring MOSAIC remained responsive to student needs. Importantly, students and faculty mentors reported an improved sense of community, belonging, and mutual support, directly addressing core needs initially identified in the design phase.

## Sustainability of MOSAIC over 5 years

MOSAIC is designed as a vehicle for institutional change, influencing mentorship policies, faculty engagement, and student support structures across Columbia Mailman School of Public Health. The need for MOSAIC programming was evident through its rapid expansion from efforts within a single department to a school wide initiative. The first steps in ensuring the sustainability of MOSAIC were through allocating necessary resources, ensuring adequate financial and administrative support, and developing a clear monitoring and evaluation plan to establish metrics and to assess the effectiveness of MOSAIC.

Following the initial year, the co-founders and directors were compensated for the considerable time spent on MOSAIC and student mentorship. Initially this program and its co-founders and directors were funded through internal grant mechanisms at the school, and then as the program grew and its importance understood the program was able to secure centralized school financial support. This centralized school support includes a budget for 10% faculty coverage for each of the two faculty co-directors, 5% faculty compensation for each department faculty affiliate, administrative support (25% of a administrative staff member), and a budget for events (including food and supplies). Securing core funding is a crucial step to institutionalizing a program like MOSAIC and demonstrates the commitment to mentorship on a school level. After the first year, as MOSAIC continued to grow, it was critical to create lasting relationships with existing resources that already existed across the school.

Program founders wanted to ensure not only that the resources and offices existed but that our students had access and were aware of these resources. These offices and resources included the office of career services, office of field practice, and office of student support including academic specialists. MOSAIC built partnerships with these offices to co-sponsor events for students including resume workshops, interview prep, APEX and job search panels, alumni panels and more. It was evident from the needs assessment stage that students were often unaware of the resources that were available to them and MOSAIC acts as a critical bridge to those existing resources.

After the initial year, it was evident that additional faculty mentors would be required and faculty mentors were then recruited from each department within the Mailman School of Public Health, carefully selected for their commitment to mentorship and equity and expertise in racism or social inequalities and population health. One mentor from each of the departments was identified and recruited with the help of the Department Chair in each department. These mentors received some training in culturally responsive mentorship approaches to better support MOSAIC mentees and are also compensated for their time. As previous explained, faculty affiliates participate in monthly MOSAIC faculty meetings for program and event planning, attend MOSAIC events, and hold monthly office hours for MOSAIC students to opt into. The model of faculty mentorship has shifted over time as the program has grown, and we have needed to adapt to the large number of students. Most recently, we added faculty affiliate office hours with smaller groups of students. All MOSAIC faculty affiliates hold these monthly office hours, and students are able to attend as many of them as they would like – providing more opportunities for networking and smaller group mentorship for our students.

The exponential growth (e.g., 26 MOSAIC students in 2019 and 453 MOSAIC students in 2024) and sustainability of MOSAIC at Columbia Mailman is largely attributed to the commitment of faculty, staff, and administration in supporting the program. Faculty members recognize the value of structured mentorship in shaping the next generation of public health professionals and ensuring that URM have the resources needed to thrive. Faculty from across all six Mailman departments participate in the program with support from all six department chairs and school leadership which signals the importance of MOSAIC and commitment to the program and the community. Staff involvement has also been instrumental in sustaining MOSAIC, particularly through program coordination, student outreach, and event planning. Student affairs offices, career services, and inclusive excellence offices have partnered with MOSAIC to expand access to institutional resources, ensuring students receive comprehensive support beyond the classroom.

At the administrative level, MOSAIC has been recognized as a key driver of institutional commitment to inclusive excellence. This is in part due to efforts to conduct ongoing research and evaluation on MOSAIC. MOSAIC is formally evaluated every other year with a mixed-methods evaluation including questions about motivations for joining, needs met and unmet and recommendations for improvements. Columbia Mailman leadership has integrated MOSAIC into broader inclusion efforts, guaranteeing funding annually since 2020. The administrative leadership is supportive of securing funding for program expansion, mentorship training, student-led initiatives, and MOSAIC-led school-wide days of service to the greater community. This institutional backing underscores the value of mentorship as a strategic priority in advancing public health education and professional development. The program has also contributed to a cultural shift within Columbia Mailman, where mentorship is increasingly recognized as a core component of public health student success. By embedding MOSAIC within the institution’s fabric, public health education becomes more inclusive and aligned with the principles of social justice.

## Conclusion

MOSAIC serves as a replicable mentorship program for other schools of public health seeking to institutionalize mentorship as a mechanism for URM student success. Key lessons from Columbia Mailman’s experience emphasize the importance of institutional commitment, student-driven program design, culturally responsive mentorship, and extending mentorship beyond the classroom. Dedicated funding, administrative buy-in, and faculty engagement are essential for sustaining mentorship programs over an initial year or funding source. Actively involving URM students in program development ensures that mentorship initiatives address their real needs and experiences. Training faculty in AOP and culturally competent mentorship strategies strengthens relationships and enhances their ability to provide student support. Additionally, integrating career development, mental health resources, and community-building initiatives into mentorship programs enhances their overall impact.

MOSAIC at Columbia Mailman demonstrates how structured faculty-to-student mentorship programs can be implemented at programs and schools of public health through an approach of fostering inclusivity and professional development. By prioritizing mentorship as an institutional commitment, public health schools can better support BIPOC and first-generation students, ultimately strengthening the field’s workforce diversity and capacity to address health disparities. The success and sustainability of MOSAIC underscore the necessity of investing in mentorship programs as a strategic imperative for student success and institutional transformation. Moving forward, public health institutions must continue to build sustainable mentorship structures that empower students and uphold the values of social justice and health equity.

## Data Availability

The original contributions presented in the study are included in the article/supplementary material, further inquiries can be directed to the corresponding author.
